# Direct-to-Caregivers Research Dissemination: A Novel Approach to Targeting End-Users

**DOI:** 10.1093/geroni/igab046.2957

**Published:** 2021-12-17

**Authors:** Lee Lindquist, Anna Liggett, Vanessa Ramirez-Zohfeld, Shahla Baharlou

**Affiliations:** 1 Northwestern University Feinberg School of Medicine, Chicago, Illinois, United States; 2 Northwestern University Feinberg School of Medicine, Northwestern University Feinberg School of Medicine, Illinois, United States; 3 Icahn School of Medicine at Mount Sinai, Icahn School of Medicine at Mount Sinai, New York, United States

## Abstract

Dissemination of geriatrics research usually occurs through conference presentations or publications viewed by colleagues in the same field. Older adults and their family caregivers have limited direct access to research findings. We sought to pilot a direct-to-caregiver workshop with the intent to disseminate geriatrics research directly to family caregivers of older adults. As part of an academic national conference, an ‘Updates in Geriatrics Research’ workshop is presented as a compilation of innovative research published in the prior year. We distilled workshop content into a lay format which was presented to family caregivers at two community-based caregiver symposiums. Mixed method surveys were completed by family caregiver attendees with open-ended responses analyzed using content and constant-comparative techniques. Of the 29 survey respondents, all were female, mean age 58.9 yrs. (range 52-72), providing care to older adults, mean age 87.2 years (range 66-97). Respondents unanimously identified learning information pertinent to their care recipient. When asked: Do you feel that direct-to-caregiver research dissemination is useful, all respondents selected yes. Open-ended responses for reasons why revealed two main themes: 1.) Creating informed caregivers: “Caregivers need this information in their toolbox.” and 2.) Empowering caregiver-advocates: “The more we know, the better we can advocate for our loved ones and challenge their health care.” Respondents all planned on sharing information with others, specifically family, friends, and physicians. In conclusion, disseminating geriatrics research direct-to-caregivers is feasible. Researchers, who present their work for scientific conferences, should consider translating their findings into presentations for community-based family caregivers.

